# Clinical characteristics and patient treatment satisfaction with Humalog U-200 in patients with type 2 diabetes mellitus: an observational study

**DOI:** 10.1080/21556660.2019.1704415

**Published:** 2019-12-20

**Authors:** Jil Mamza, Uchena Anyanwagu, Mohammed Alkharaiji, Iskandar Idris

**Affiliations:** Division of Medical Sciences and Graduate Entry Medicine, School of Medicine, University of Nottingham, Royal Derby Hospital, Derby, UK

**Keywords:** Humalog U-200, KwikPen, treatment satisfaction, clinical characteristics, real-world

## Abstract

**Background:**

There are limited data on the real-world evidence of Humalog 200 units/ml KwikPen (U-200) insulin. We assessed the use of U-200 insulin in UK routine clinical practice to provide information on clinical characteristics, treatment satisfaction and short-term clinical outcomes.

**Methods:**

Nine patients with type 2 diabetes who initiated U-200 in secondary care and a further 12 identified from primary care electronic database were enrolled. A treatment satisfaction questionnaire was administered to the 19 secondary care patients. Follow-up data on clinical parameters were collected at 3 and 6 months following initial U-200 insulin administration and the data were used to assess changes in clinical outcomes from baseline.

**Results:**

Secondary care patients had a mean age 60 ± 11 years, mean HbA1c of 8.6% ± 1.3% and a mean BMI of 39.7 ± 5.3 kg/m^2^ at baseline. Primary care database patients had a mean age 57 ± 13 years, mean HbA1c 10.3% ± 1.7 and a mean BMI 42.3 ± 3.8 kg/m^2^. The nine participants’ responses to the questionnaire suggested a high preference for U-200 over a previous mealtime insulin pen (PMIP). On average, the patients agreed that U-200 was quicker to inject, had a better controlled home blood glucose reading and less discomfort at the injection site compared to a PMIP. Patients were willing to continue with their U-200 treatment. No significant HbA1c reduction was observed at 3 months in the secondary care group (−0.5%), but marked significant reduction in HbA1c was seen at 3 months in the primary care dataset to (−2.8%; *p* < .0004). There was also some suggestion of weight loss in both the secondary and primary care groups.

**Conclusion:**

Humalog U-200 insulin users were comprised mainly of older patients with diabetes complications and high HbA1c levels at the time of U-200 initiation. Overall, U-200 improved patients’ satisfaction with diabetes treatment and short-term metabolic outcomes.

## Introduction

Insulin lispro 200 units/ml (Humalog U-200 KwikPen) is a concentrated rapid-acting insulin analog bio- and therapeutically equivalent to the 100 units/ml Humalog formulation^1^. However, U-200 KwikPen holds 600 total units of insulin in the same sized pen that previously could only hold 300 total units. Specific advantages of U-200 compared with conventional U-100 insulin include less volume for injection resulting in an easier glide force for injection^2^. Given the importance of simplifying insulin injection^3^ by using less volume and less force required for injection, it is hypothesized that the use of Humalog U-200 may contribute to improving treatment adherence and, consequently, metabolic control. Additionally, since each pen last longer, patients will not be required to request repeat prescription as frequently, which would ensure less risks of missing injections due to pen running out of insulin. Although, Humalog U-200 is a practical alternative regimen to people with diabetes who take higher doses of rapid-acting (e.g. >0.5 units/kg), post-marketing surveillance or real-world evidence assessing the use of U-200 insulin in routine practice is lacking.

Factors that may explain insulin treatment failure, include complex diabetes regime, polypharmacy, side effects, therapeutic inertia and non-adherence^4^. In addition, incorrect insulin administration techniques and pen misuse^5^ may greatly affect metabolic control in patients on multiple daily injections. Routine clinical practice provides a useful source of data for evaluating clinical effectiveness, but to date, no real-world study in the UK has described the clinical characteristics of patients administering Humalog U-200 and the extent to which patients are satisfied with their diabetes treatment. Therefore, we examined the clinical characteristics of patients who were administered Humalog U-200 insulin in routine clinical practice and to describe using questionnaires, patients’ perceptions of their diabetes treatment following the use of Humalog U-200 KwikPen, compared with their previous mealtime insulin pen (PMIP) of >3 months duration. The study also used anonymized health records of randomly recruited patients in UK primary care between July and September 2017. In addition, the study analyzed their follow-up clinical parameters’ data at 3 and 6 months following initial administration.

## Methods

### Study design and setting

This was an observational study using anonymized medical records of adult patients (aged >18 years) who administered Humalog U-200 in UK clinical practice. Clinical parameters of patients were collected from five secondary care centers in the UK. Follow-up data on clinical parameters were collected at 3 and 6 months following initial Humalog U-200 administration. A further 12 patients who were commenced on Humalog U-200 was identified from the Health Improvement Network, a primary care electronic database. A questionnaire was administered to nine individual patients by post or by face-to-face meeting to investigate their experience of using Humalog U-200 and their satisfaction with their diabetes treatment following U-200 treatment initiation. Patients did not receive any reimbursement for their contribution to the study.

### Demographic and clinical parameters

The baseline demographic and clinical parameters were selected *a priori* on the basis of clinical significance and included in the descriptive analyses. These include age at initial U-200 treatment, gender, ethnic background, type of diabetes, duration of diabetes, body weight and medications were included in the data collection. Microsoft Access was used to collect anonymized data from secondary care patients. Information on complications such as stroke, peripheral artery disease, retinopathy, nephropathy and neuropathy were also assessed. Data were collated on clinical parameters at baseline, at the third and at the sixth months following the commencement of U-200 insulin. Information on patients’ antidiabetic therapy including insulin administration at baseline, and at the third and sixth months following U-200 treatment initiation were assessed. Comparison of the changes in patients’ glycemic hemoglobin (HbA1c) and weight, body mass index (BMI), at baseline and at 3 and 6 months post-Humalog U-200 insulin treatment initiation were calculated.

### Treatment experience and satisfaction survey

The patient confidence and satisfaction survey were designed by the current study investigators in accordance with NHS guidelines or recommendations available from www.nhssurveys.org. Participants completed this by mail at a minimum of 3 months after starting U-200. The questionnaire comprised two main sections. The first (Q2) described short statements relating to the patients’ experience of the Humalog U-200 in comparison to their PMIP following the commencement of Humalog U-200. The second (Q3) section described statements relating to patients’ satisfaction with their diabetes treatment following the initiation of Humalog U-200 insulin treatment. For each of the statements, patients were asked to rate their level of preference by choosing from a 5-point Likert scale option ranging from “strongly disagree” to “strongly agree”. Data on individual patient’s response to the survey were collected using paper questionnaires. The survey information and descriptions of statements are included in Supporting Information I.

### Data analyses

The characteristics and clinical parameters of the patients are summarized using descriptive statistics. A simple paired *t*-test was used to measure change in the means HbA1c and weight at 3 months and 6 months after administering Humalog U-200 compared to the baseline measurements. Analysis was conducted separately for primary care and secondary care cohort due to different information being available for both cohorts and because the main of this work is to explore treatment satisfaction outcomes The variability in the extent to which patient responded to the questionnaires was assessed across each of the statements presented to the patients in the survey. Threshold for statistical significance was *p* < .05. Statistical calculations were performed using Stata 14.

### Details of ethics approval

The study was approved by London – West London & GTAC Research Ethics Committee.

## Results

### Clinical characteristics

Table S1 (see Supplementary Appendix 3) summarizes the baseline values of secondary care patient and clinical characteristics for nine patients whose medical care records were collected. All the patients were Caucasian with a mean age of approximately 60 ± 11) years, and comprised of predominantly men (89%, *n* = 8). The average HbA1c at baseline was 8.6% ± 1.3% and their average BMI was 39.7 ± 5.3) kg/m^2^ at baseline. Over half of the patients (56%) had a known diabetes complication at baseline and 22% had hypoglycemia unawareness. Specific complications recorded at baseline include CHD (22%), retinopathy (44%), nephropathy (56%) and neuropathy (44%). Thirty-three percent of patients received a intermediate and long-acting insulin, whereas 22% received a short-acting insulin therapy as their first insulin regimen. The remaining patients received biphasic insulin previously. Forty-four percent administered U-200 insulin three times daily on commencement. There was one patient recorded to have had U-200 insulin once a day and another one patient recorded to have had U-200 up to four times a day. Overall, the average daily dose of U-200 insulin was approximately 154.3 ± 104.1.

Within the primary care dataset, 3 out of the 12 who were using Humalog U-200 KwikPen were male (25%). The mean age at baseline was 57.0 ± 12.9. The average HbA1c at baseline 10.3% ± 1.7 and an average BMI of 42.3 ± 3.8 kg/m^2^. Seven patients were found to be using rapid-acting insulin prior to the introduction of Humalog U-200. Four of these patients used rapid-acting insulin as part of a basal-bolus regimen.

### User experience and satisfaction

**Figure 1.**
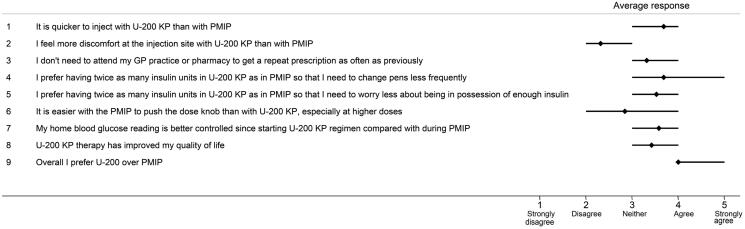
Response to questionnaire on user experience survey (Supplementary Appendix 1). It shows the variability in the response scores given by all nine respondents for the statements in Supplementary Appendix 1 of the questionnaire in Supplementary Information I. The range plots (horizontal bars) represent the 25th and 75th percentile of the mean score. Abbreviations. U-200 KP, Humalog 200 units/ml KwikPen; PMIP, previous mealtime insulin pen.

**Figure 2.**
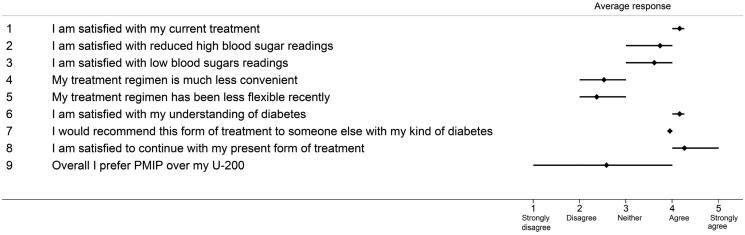
Patient satisfaction of diabetes treatment using U-200 insulin (Supplementary Appendix 2). It shows the variability in the response scores given by all nine respondents for the statements in Supplementary Appendix 2 of the questionnaire in Supplementary Information I. The range plots (horizontal bars) represent the 25th and 75th percentile of the mean score. Abbreviations. U-200 KP, Humalog 200 units/ml KwikPen; PMIP, previous mealtime insulin pen.

Figure 1 illustrates the variability in the experiences of patients who used Humalog U-200 KwikPen. Overall, the patients’ responses to the questionnaire showed a high preference for Humalog U-200 KwikPen over a PMIP. Patients found it quicker to inject with U-200 KwikPen than with a PMIP and perceived their home blood glucose reading was better controlled since starting Humalog U-200 KwikPen. Figure 2 describes the differences in the extent to which patients were satisfied with their diabetes treatment after initiating therapy with U-200 KwikPen. The extent to which patients were satisfied with their current diabetes treatment was on average 4.2 on a 5-point scale, and were satisfied to continue with their present form of treatment (average score = 4.3).

### Short-term clinical outcomes

#### HbA1c

In the secondary care group, the average HbA1c was lower at all time points during the follow-up period compared to baseline; with no statistically significant difference. At 3 months, the mean HbA1c had reduced by 0.5% compared to the baseline, and the reduction remained at 0.5% at 6 months (*p* value = .5).

In the primary care dataset, HbA1c was lower at all time points during the follow-up period compared to baseline; with only a statistically significant difference at the 3-month stage. At 3 months, the mean HbA1c was 7.5% ± 1.5 with a reduction of −2.8%l compared to the baseline (*p* = .0004). Mean HbA1c at 6 months was 9.0% ± 0.4 (*p* = .094)

#### Body weight

In the secondary care group, the mean weight at baseline was 117.9 (SD ± 18.3) kg and the calculated mean BMI was 38.7 ± 5.3 kg/m^2^. Mean weight at 6 months was 104.3 ± 4 kg/m^2^. This was observed to be 13.6 kg lower at 6 months (there was no weight measurement at 3 months) compared to the baseline, however, the reduction in body weight was not statistically significant.

In the primary care dataset, the mean weight at baseline is 117.1 ± 11.1 kg and the calculated mean BMI was 42.3 ± 3.9 kg/m^2^. Both weight and BMI were found to be lower at all time points from baseline onwards, with only one statistically significant difference at 3 months. The mean weight at 3 months 101.2 ± 16.9 kg revealed a reduction of 16.0 kg compared to the baseline (*p* = .007). Mean weight at 6 months, however, was 111.7 (*p* = .54).

## Discussion

### Main findings

On average, the patients perceived U-200 insulin was quicker to inject, had a better controlled home blood glucose reading and less discomfort at the injection site compared to a PMIP. The patients’ response to the questionnaire suggested they had a strong preference for U-200 over a PMIP and were willing to continue with their U-200 treatment. Metabolic results from this study also indicate a short-term benefit of U-200 in reducing HbA1c and other clinical parameters of interest including weight. Whether the reduction in weight induced reduction on Hba1c or vice versa is unclear and is outside the remit of this study, but we believe these parameters are codependent.

Our findings support findings from a previous study from Italy which indicated a positive treatment satisfaction from most patients, which translated to improved short-term HbA1c levels. Interestingly, in that study, there was a dosage reduction of Humalog U-200 and reduction in the risk of hypoglycemia^6^. Our study did not show this, likely due to the small number of patients who use this insulin formulation. Nonetheless, the advantages of less injection effort with Humalog U-200 in our study support previous studies where volume reduction will result in less force to be applied to the pen^7–9^. This may translate to improved HbA1c level, although such broad conclusions cannot be made from our study due to the small number. However, due to the high prevalence of limited joint mobility in patients with diabetes^10^^,^^11^, the ease of injection may help many patients to deliver their insulin more safely and effectively. Furthermore, it is tempting to speculate that less injection volumes will prevent wastage of insulin, conferring long-term economic benefits.

### Strengths and limitations

To our knowledge, this study is the first observational study on the clinical outcomes of Humalog U-200 KwikPen in the UK. This study is strengthened by data providing real-world evidence from routine clinical care. Given that this study examined short-term clinical outcomes, it is understandable that the patient who initiated insulin treatment with Humalog U-200 will have very high expectations for their metabolic outcomes. Of note, however, these patients are older with high Hba1c at baseline – suggesting more complicated patients with type 2 diabetes. Major limitations of this study include the short duration of follow-up observations and the small number of patients involved. Another limitation includes the possibility that the questionnaires may not have accounted for the entire experience a patient might have had while using U-200 KwikPen, due to recall bias, the fact that these are un-validated questionnaires and lack of information of fasting and post-prandial glucose hemodynamics as well as hypoglycemia events. Nevertheless, preliminary findings from the study show that Humalog U-200 reduced, HbA1c and possibly weight within 3–6 months of treatment initiation and such favorable metabolic results in combination with ease of use provide objective support to patients’ preference ratings.

## Conclusion

To conclude, Humalog U-200 is an appropriate therapeutic option for patients with type 2 diabetes who require insulin treatment to manage their hyperglycemia. Its use was associated with short-term reduction in Hba1c and weight with favorable effects on treatment satisfaction. The use of lower injection volume and ease of slide may benefit patients with limited joint mobility or those who are on high doses of insulin. This study also underscores the importance of further studies in clinical decision-making on individualized pen devices to improve treatment compliance and glycemic control.

## Supplementary Material

Supplemental Material

## Transparency

### Declaration of financial/other relationships

I.I. has received funding from Eli Lilly for lecture fees and research grants. I.I. did not receive any personal fees for his involvement in this study. The remaining authors and JDA peer reviewers on this manuscript have no relevant financial or other relationships to disclose.

## References

[1] AIFA (Italian Drug Agency) Nota Informativa Importante su Humalog KwikPen. [cited 2016 May 25] Available from: http://www.agenziafarmaco.gov.it/content/nota-informativa-importante-su-humalogkwikpen-25052016

[2] Rees TM, Lennartz AH, Ignaut DA. A comparison of glide force characteristics between 2 prefilled insulin lispro pens. J Diabetes Sci Technol. 2015;9(2):316–319. 25591858 10.1177/1932296814567533 PMC4604579

[3] Clapham L. Injection technique education and follow-up: the key to ensuring optimal glycaemic control. J Diabetes Nurs. 2015;19:152–155.

[4] Gentile S, Ceriello A, Pipicelli G, et al. Type 2 diabetes mellitus treatment habits in a specialized care setting: the START-DIAB study. Mediterranean J Nutr Metab. 2017;10(2):165–179.

[5] Gentile S, Strollo F, De Rosa N, et al. Injection-related local side effects in the treatment of diabetes mellitus: a methodological approach and possible solutions. Consensus statement of AMD-OSDI study group on injection technique. 2016 Diabetes complications. Dover (DE): SM Group; [cited 2019 Oct 28]. Available from: http://www.smgebooks.com/diabetic-complications/chapters/DC-16-04.pdf

[6] Gentile S, Fusco A, Colarusso S, et al. A randomized, open-label, comparative, crossover trial on preference, efficacy, and safety profiles of lispro insulin u-100 versus concentrated lispro insulin u-200 in patients with type 2 diabetes mellitus: a possible contribution to greater treatment adherence. Expert Opin Drug Saf. 2018;17:445–450. 29564932 10.1080/14740338.2018.1453495

[7] Rissler J, Jørgensen C, Rye Hansen M, et al. Evaluation of the injection force dynamics of a modified prefilled insulin pen. Expert Opin Pharmacother. 2008;9(13):2217–2222. 18710347 10.1517/14656566.9.13.2217

[8] Asakura T, Seino H, Kageyama M, et al. Evaluation of injection force of three insulin delivery pens. Expert Opin Pharmacother. 2009;10(9):1389–1393. 19466910 10.1517/14656560903018929

[9] Owens DR. Study to compare the injection force required for the following insulin pen devices: Lilly disposable pen, Novo FlexPen, and Solostar. J Diabetes Sci Technol. 2007;1:A134.

[10] Shinabarger NI. Limited joint mobility in adults with diabetes mellitus. Phys Ther. 1987;67(2):215–218. 3809247 10.1093/ptj/67.2.215

[11] Gerrits GE, Landman GW, Nijenhuis-Rosien L, et al. Limited joint mobility syndrome in diabetes mellitus: a minireview. World J Diabetes. 2015;6(9):1108–1112. 26265997 10.4239/wjd.v6.i9.1108 PMC4530324

