# Supplementary material for: Clinical characteristics and patient treatment satisfaction with Humalog U-200 in patients with type 2 diabetes mellitus: an observational study
Source: J Drug Assess. 2019 Dec 20;9(1):8–12. doi: 10.1080/21556660.2019.1704415 (PMC6968701; doi:10.1080/21556660.2019.1704415)
Supplement: Supplemental Material [file IJDA_A_1704415_SM8938.docx]

**Supplementary Information I**


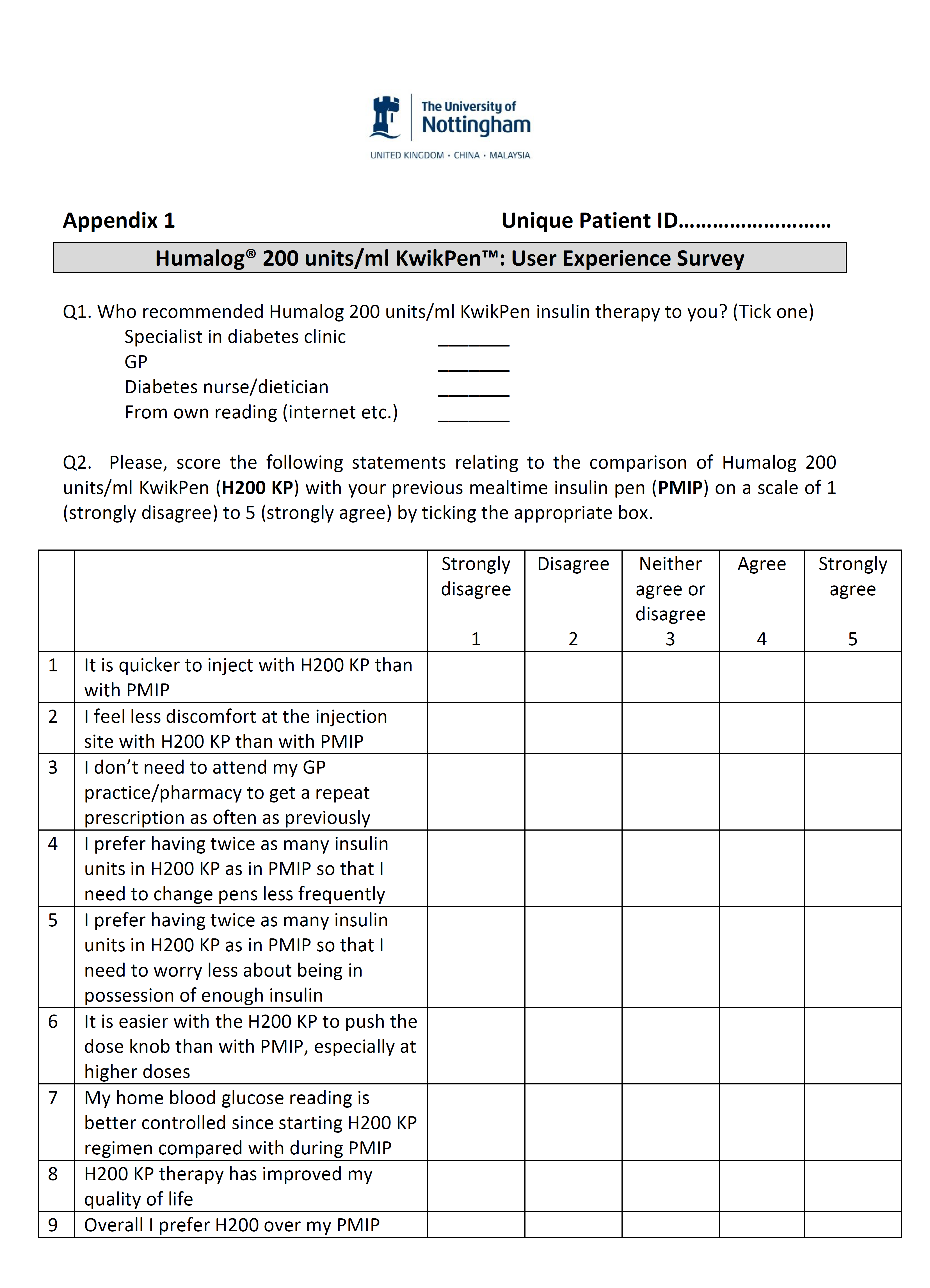


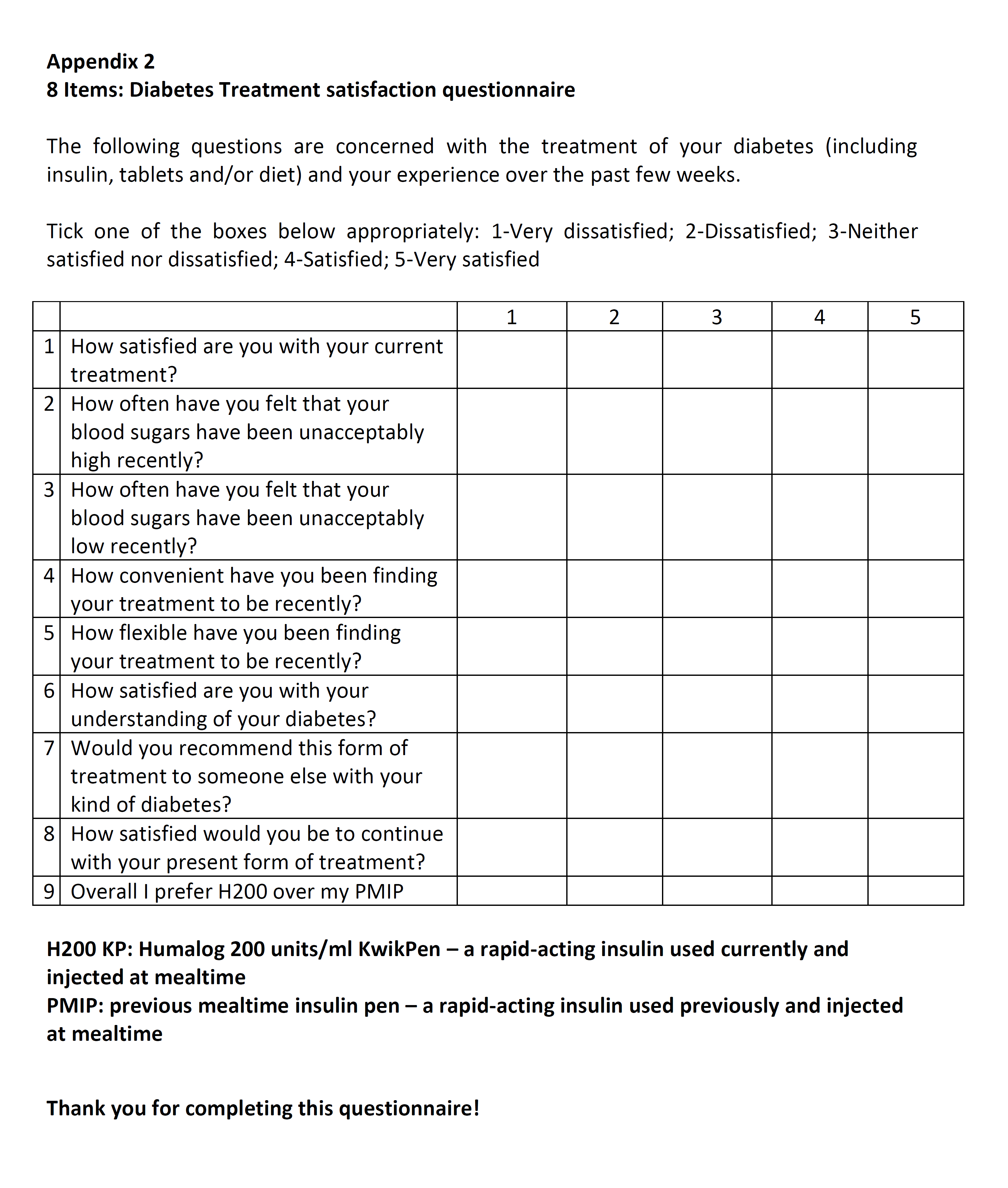


**Appendix 3**

**Table 1: Baseline patient characteristics for the secondary care cohort**

| **Characteristics** | **Mean ± SD or N (%)** |
| --- | --- |
| Overall, N | 9 (100%) |
| **Demographics** |  |
| Age, years | 59.8 ± 10.7 |
| Gender |  |
| Female | 1 (11.1%) |
| Male | 8 (88.9%) |
| Ethnicity |  |
| White | 9 (100.0%) |
| Diabetes duration, years | 20.8 ± 11.6 |
| Regular exercise | 1 (11.1%) |
| **Comorbidities** |  |
| Diabetes complication | 5 (55.6%) |
| Hypoglycaemia unawareness | 2 (22.2%) |
| CHD | 2 (22.2%) |
| Retinopathy | 4 (44.4%) |
| Nephropathy | 5 (55.6%) |
| Neuropathy | 4 (44.4%) |
| **Metabolic parameters** |  |
| HbA1c (%) | 8.6 ± 1.3 |
| Total cholesterol (mmol/L) | 3.9 ± 0.5 |
| Triglyceride () | 2.3 ± 0.8 |
| LDLC (mmol/L) | 1.9 ± 0.4 |
| HDLC (mmol/L) | 1.0 ± 0.2 |
| Body weight (kg) | 117.9 ± 18.3 |
| BMI (kg/m^2^) | 38.7 ± 5.3 |
| ACR | 3.4 ± 1.6 |
| eGFR (mL/min/1.73 m²) | 62.4 ± 16.6 |
| SBP (mmHg) | 144.2 ± 18.6 |
| DBP (mmHg) | 76.4 ± 10.7 |
| **Diabetes treatments** |  |
| First insulin administered | |
| Humalog (basal/bolus) | 1 (11.1%) |
| Novorapid ( basal/boluses) | 1 (11.1%) |
| Humalog mix 25 | 1 (11.1%) |
| Humulin I | 1 (11.1%) |
| Insuman Comb 25 | 1 (11.1%) |
| Missing | 4 (44.4%) |
| Type of first insulin administered | |
| Medium and long-acting | 3 (33.3%) |
| Short-acting | 2 (22.2%) |
| Missing | 4 (44.4%) |
| Continuing other GLT | 3 (33.3%) |
| Total daily dose of U-200 | 154.3 ± 104.1 |
| Frequency of U-200 administration | |
| Once Daily | 1 (11.1%) |
| Three Times Daily | 4 (44.4%) |
| Four Times Daily | 1 (11.1%) |
| Missing | 3 (33.3%) |

Values are quoted as actual numbers (%) or as mean (standard deviation).
